# Inducible CreER^T2^ Mouse Lines for Characterization of Retinal Bipolar Cell Subtypes

**DOI:** 10.1523/ENEURO.0483-25.2026

**Published:** 2026-04-16

**Authors:** Ebenezer J. Quainoo, Xiaoling Xie, Lotem Kol, Joseph Samson, Lin Gan

**Affiliations:** ^1^Department of Neuroscience and Regenerative Medicine, Medical College of Georgia, Augusta University, Augusta, Georgia 30912; ^2^James and Jean Culver Vision Discovery Institute, Medical College of Georgia, Augusta University, Augusta, Georgia 30912; ^3^Department of Medical Illustration, College of Health and Allied Sciences, Augusta University, Augusta, Georgia 30912

**Keywords:** bipolar cells, genetic tools, inducible Cre, retina, subtype characterization

## Abstract

Bipolar cells relay visual signals from photoreceptors to ganglion cells. In the mouse retina, 15 bipolar cell subtypes have been identified and are classified as ON or OFF bipolar cells based on their responses to light or as rod or cone bipolar cells based on their photoreceptor connectivity. Despite this diversity, the distinct structural and functional roles of bipolar cell subtypes in visual information processing remain poorly understood, largely due to lack of tools and models for their characterization. In this study, we generated inducible Cre mouse lines driven by the promoters of *Vsx1*, *Lhx3*, and *Lhx4* and crossed them with ChR2EYFP reporter mice to trace lineage and characterize bipolar cell subtypes in postnatal and adult mouse retinas. Following tamoxifen induction in adult male and female mice, ChR2EYFP expression was detected in type 2, 6, and 7 bipolar cells in the *Vsx1*CreER^T2^ line; type 1b, 2, and 6 bipolar cells in the *Lhx3*CreER^T2^ line; and type 2, 3, 4, and 5 bipolar cells in the *Lhx4*CreER^T2^ line. In addition, *Lhx4*CreER^T2^ activity was observed in cone photoreceptor cells. ChR2EYFP expression was also detected in other ON and OFF cone bipolar cells, as well as rod bipolar cells, when tamoxifen induction was performed in the postnatal mice. These inducible Cre lines enable genetic manipulation in retinal bipolar cell subtypes at different developmental time points and serve as tools for elucidation of the mechanisms that control bipolar cell subtype development and function.

## Significance Statement

Bipolar cells are central connectors of the outer and inner retina and initiate processing of complex visual information. Bipolar cells differentiate into more than 15 subtypes during development, and their structural diversity has been well studied. However, the unique contribution of these subtypes to visual information processing is poorly understood due to inadequate tools. In this study, we develop and characterize three inducible Cre mouse models for temporally defined genetic manipulation in bipolar cell subtypes in the developing and adult mouse retina. These models serve as tools to elucidate the mechanisms that regulate development and function of bipolar cell subtypes in the mouse retina.

## Introduction

**Figure 1.**
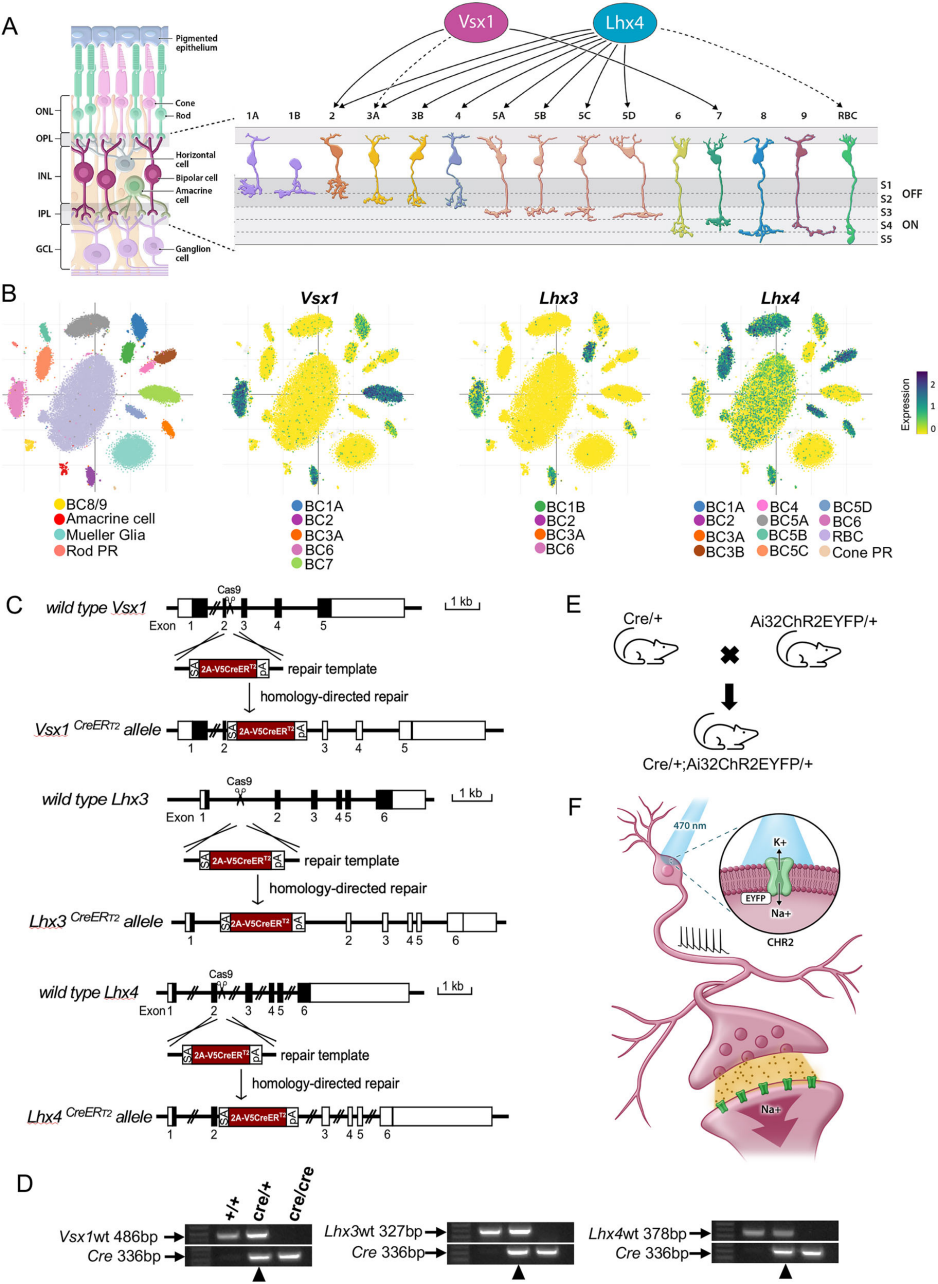
Bipolar cell subtype-specific CreER^T2^ mouse lines. ***A***, Schematic of retinal cross section highlighting 15 bipolar cell subtypes in the inner nuclear layer (INL); including type 1–4 (OFF bipolar cells) with axon terminal stratification in sublaminae 1 and 2, and type 5–9 as well as rod bipolar cell (ON bipolar cells) with axon terminal stratification in sublaminae 3, 4, and 5 in the inner plexiform layer (IPL). *Vsx1* is expressed in type 2 and 7 bipolar cells and transiently expressed (dashed lines) in type 3a bipolar cells (Shi et al., 2011, 2012). *Lhx4* is also expressed in type 2, 3, 4, and 5 bipolar cells and transiently expressed in rod bipolar cells (Dong et al., 2020). ONL, outer nuclear layer; OPL, outer plexiform layer; GCL, ganglion cell layer; RBC, rod bipolar cell; S, sublaminae. ***B***, Scatterplot of bipolar cell single-cell RNA sequencing (Shekhar et al., 2016) showing *Vsx1* expression in type 1a, 2, 3a, 6, and 7 bipolar cells; *Lhx3* expression in type 1b, 2, 3a, and 6 bipolar cells; and *Lhx4* expression in type 1a, 2, 3, 4, 5, 6, and rod bipolar cells as well as cone photoreceptors in P17 mouse retina. ***C***, Schematic diagram of CreER^T2^ knock-in into *Vsx1*, *Lhx3*, and *Lhx4* utilizing CRISPR-Cas9-mediated homology-directed repair. CreER^T2^ knock-in allele contains a splicing acceptor site (SA), P2A peptide, V5 tag, and a polyA (PA) for transcription termination. ***D***, Genotyping of *Vsx1*, *Lhx3*, and *Lhx4* wild-type, heterozygous, and homozygous CreER^T2^ knock-in mice. Heterozygous knock-in mice (black arrowhead) were selected for characterization. ***E***, Schematic of CreER^T2^ knock-in mice cross to Ai32ChR2EYFP mice to generate double-heterozygous CreER^T2^/+;Ai32ChR2EYFP/+ mice. ***F***, Illustration of light-induced opening of ChR2 and subsequent initiation of electrical activity in a bipolar interneuron.

The retina is a light-sensitive neural tissue composed of neuronal and supporting glial cells arranged in a laminar structure. Photoreceptor cells, as modified sensory neurons, transduce light stimuli for processing and transmission through horizontal, bipolar, amacrine, and retinal ganglion cells to be conveyed to visual processing centers in the brain (Fig. 1*A*).

Bipolar cells are first-order interneurons that connect, process, and relay visual information from the outer retina to the inner retina. Their cell bodies lie in the outer part of the inner nuclear layer with dendrites and axons extending into the outer and inner plexiform layers of the retina. Bipolar cells, like other neuronal cell types in the retina, comprise many subtypes (Boycott and Wässle, 1991; Ghosh et al., 2004; Yan et al., 2020). These subtypes are broadly categorized into ON and OFF bipolar cells depending on their response to light or rod and cone bipolar cells depending on the type of photoreceptor cell inputs they receive (Masu et al., 1995; Haverkamp et al., 2000; Hack et al., 2001; Tsukamoto et al., 2001). Ghosh et al. (2004) further classified ON and OFF bipolar cells based on the level of their axon terminal stratification (Fig. 1*A*). OFF bipolar cell axons stratify in sublaminae 1 and 2, and ON bipolar cell axons stratify in sublaminae 3, 4, and 5 in the inner plexiform layer of the retina. Bipolar cells have also been classified based on their gene expression profile, revealing 15 distinct subtypes that correspond to their morphological classification (Shekhar et al., 2016). Notably, neuronal subtype classification based on their gene expression profile gives significant insights into the regulatory mechanisms that control subtype formation, differentiation, and function.

All 15 bipolar cell subtypes make connections to other neuronal cell types in the retina and form the initial processing center of the visual system. However, the specific factors that control bipolar cell subtype specification and differentiation during retinal development have not been well described. The distinct functions of these subtypes, that is, the role of each subtype or group of subtypes in visual information processing is also not well defined. Although, contacts made by these subtypes to groups of photoreceptor cells and amacrine cells infer their function (Euler et al., 2014; Behrens et al., 2016; Tsukamoto and Omi, 2017). This warrants the development of tools, models, and techniques for further study of bipolar cell development and function.

During retinogenesis, homeobox genes encode homeodomain-containing transcription factors that carry out essential roles in cell specification and differentiation in the retina (Zagozewski et al., 2014). Among these transcription factors are VSX1, LHX3, and LHX4 which specifically regulate bipolar cell subtype development in the retina (West and Cepko, 2022; Quainoo and Gan, 2025). *Vsx1* has been shown to be expressed in type 7 bipolar cells and OFF bipolar cell subtypes that coexpress type 2 bipolar cell markers in adult GUS8.4GFP and *Vsx1*:^t^lacZ transgenic reporter mice (Shi et al., 2011; 2012). Transient expression of *Vsx1* is also detected in type 3a OFF bipolar cells during the first two postnatal weeks in the mouse retina (Shi et al., 2012). *Lhx4* expression, on the other hand, has been reported in OFF type 2, 3, and 4 bipolar cells and ON type 5 bipolar cells, with transient expression in rod bipolar cells during the first two postnatal weeks in the mouse retina (Dong et al., 2019; 2020; Fig. 1*A*).

Shekhar et al. (2016) further characterized the gene expression profiles of retinal bipolar cell subtypes in P17 mouse retinas and reported *Vsx1*, *Lhx3*, and *Lhx4* expression in both ON and OFF bipolar cells. Single-cell RNA sequencing data from this study show *Vsx1* expression in type 2, 3a, and 7 bipolar cells as well as in type 1a and 6 bipolar cells. *Lhx4* expression, consistent with Dong et al. (2020), is detected in type 2, 3, 4, and 5 bipolar cells and *Lhx3* expression in types 1b, 2, and 6 bipolar cells. Weak expression of *Lhx3* is also detected in type 3a bipolar cells and *Lhx4* in type 1a, type 6, and rod bipolar cells (Fig. 1*B*). These gene expression patterns demonstrate both overlapping and distinct expression of *Vsx1*, *Lhx3*, and *Lhx4* in different bipolar cell subtypes during bipolar cell development.

Transgenic Cre reporter mouse models including *Kcng4*-cre, *Neto1*-cre, *Fezf1*-cre, and *Pcp2*-cre have been used to characterize retinal bipolar cell subtypes (Zhang et al., 2004; Duan et al., 2014; Daigle et al., 2018). However, these reporter mouse models are either not inducible or not specific to retinal bipolar cells. In this study, we developed and characterized three inducible Cre mouse models using promoters of transcription factors involved in bipolar cell subtype development, namely, *Vsx1*, *Lhx3*, and *Lhx4*. Using these models, we demonstrate the expression of these transcription factors during bipolar cell development in the postnatal and adult mouse retina. These models allow precise timed manipulation of genes in specific bipolar cell subtypes for better characterization of mechanisms regulating bipolar cell subtype differentiation and function.

## Materials and Methods

### Generation of mouse lines

Mice were generated by the Augusta University Genome Editing Core using CRISPR/Cas9-based approach. Briefly, the DNA repair template containing CreER^T2^ mutations was synthesized and purified by Integrated DNA Technologies. Single-guide RNA (sgRNA) targeting *Vsx1*, *Lhx3*, and *Lhx4* gene locus was synthesized and purified by Synthego. RNP complex of 60 pmol Cas9 protein (IDT, Alt-R S.p. Cas9 Nuclease V3, stock #1081059) and 60 pmol sgRNA was formed and mixed with 300 pmol repair template DNA in 50 µl injection buffer and microinjected into fertilized eggs from C57BL/6J mice (Jackson Laboratory, stock #000664). Viable two-cell stage embryos were transferred into pseudopregnant Swiss Webster females (Taconic Biosciences; stock #SW-F-EF) to generate founder mice. The positively targeted founder mice were identified by external long-range PCRs and Sanger sequencing by Azenta Life Sciences. Founder mice were subsequently bred with wild-type C57BL/6J mice for germline transmission to generate F1 mice. F1 heterozygous pups with desired mutation were further confirmed by external long-range PCRs and Sanger sequencing. Mice were housed in a standard 12 h light/dark cycle. All animal procedures were approved by the Institutional Animal Care and Use Committee (IACUC) at Augusta University (protocol# 2019-1012) and conducted in accordance with the US National Institutes of Health Guide for the Care and Use of Laboratory Animals. All Cre knock-in mouse lines were crossed to a Cre recombinase-dependent reporter mouse line expressing ChR2EYFP from the *Rosa26* locus (Ai32; JAX Strain# 012569). Mice of both sexes were used in all experiments.

### Tamoxifen treatment

Three doses of 75 mg/kg tamoxifen were administered through intraperitoneal injection to adult *Lhx3*CreER^T2^ and *Lhx4*CreER^T2^ mice between postnatal day (P) 50–55. Retinal tissues or eyecups were harvested 1 week after final injection. Tamoxifen injections in *Vsx1*CreER^T2^ mice were administered earlier at P40 for a more efficient Cre recombination and reporter expression. For Cre induction in postnatal mice, 50 µl of 20 mg/ml tamoxifen was administered to pups through intraperitoneal injection at P3, P5, and P7, and eyecups of mice were harvested at P60.

### Immunohistochemistry

Mice were euthanized with CO_2_ and cervical dislocation. Eyeballs were enucleated and briefly fixed in ice-cold 4% PFA for 5 min, and cornea, iris, and lens were removed in 1× PBS. Eyecups were fixed in 4% PFA at 4°C overnight and thoroughly washed twice for 15 min each in 1× PBS. Washed eyecups were then dehydrated with sucrose gradient, embedded and quickly frozen in OCT for cryosectioning. Immunofluorescence staining was performed as previously described (Guo et al., 2023). Briefly, 14-µm-thick cryosections were washed in 1× PBS, followed by permeabilization in 0.3% PBST (Triton X-100 in 1× PBS). Sections were then blocked with 10% normal horse serum in 0.3% PBST (blocking solution) in a humidity chamber for 1 h at room temperature. Primary antibodies diluted in blocking solution were applied to sections and incubated overnight at 4°C. Sections were washed twice for 15 min each in 0.1% PBST after primary antibody incubation and incubated with secondary antibodies diluted in blocking solution for 1 h at room temperature. Conjugated second primary antibody was applied for overnight incubation at 4°C after washing off secondary antibody in 0.1% PBST. Sections were then washed in 0.1% PBST and 1× PBS after second primary antibody incubation and incubated in DAPI for nuclei staining for 10 min followed by mounting with coverslips after brief washing of DAPI. For whole-mount retinas, enucleated eyeballs were fixed in 4% PFA overnight at 4°C. Whole retinas were dissected and fixed in 4% PFA for 1–2 h at 4°C and then washed twice in 1× PBS for 15 min each. Immunolabeling was then performed as above, and retina was flat-mounted on glass slides for imaging. Primary antibodies used were rabbit anti-GFP Alexa Fluor 488 (1:500; Molecular Probes, #A-21311), sheep anti-VSX2 (1:200; Exalpha, #X1180P), sheep anti-calretinin (1:500; Molecular Probes, #PA5-95651), goat anti-BHLHB5 (1:1,000; Santa Cruz, #sc-6045), mouse anti-calsenilin (1:250; Millipore, #05-756), mouse anti-PKARIIb (1:500; BD Biosciences, #610625), rabbit anti-red/green opsin (1:150; Millipore, #ab5405), rabbit anti-PKCα (1:8,000; Sigma, #P-4334). Corresponding secondary antibodies were applied at a dilution of 1:1,000 and are as follows: Alexa Fluor 647-conjugated donkey anti-mouse (Thermo Fisher Scientific, #A-32787), Alexa Fluor 647-conjugated donkey anti-goat (Thermo Fisher Scientific, #A-32849), Alexa Fluor 568-conjugated donkey anti-goat (Thermo Fisher Scientific, #A-11057), Alexa Fluor 647-conjugated donkey anti-rabbit (Thermo Fisher Scientific, #A-31573), Alexa Fluor 647-conjugated donkey anti-sheep (Thermo Fisher Scientific, #A-21448), Alexa Fluor 568-conjugated donkey anti-sheep (Thermo Fisher Scientific, #A-21099).

### Imaging

Retinal sections were imaged using a Leica Stellaris confocal microscope (Leica Microsystems) with 20× or 40× oil objective lenses in the 405, 488, 568, and 647 nm laser channels. LASX software was used to obtain *Z*-stacks with a step size of 0.1–0.5 µm and processed as 2D maximum intensity projections. Images were obtained at a 1,024 × 1,024 pixel resolution with a line average of two scans. Channels were overlaid whenever appropriate, and brightness and contrast adjustments were made using Adobe Photoshop (Adobe).

### Statistics

Descriptive statistics were used to determine the distribution and density of bipolar cells in retina sections and whole mounts. EYFP-expressing bipolar cells in randomly selected 290 µm × 290 µm regions from three different retinal flat mounts were counted and averaged for quantitative analysis. Proportions were calculated by averaging counts of EYFP-expressing ON bipolar cell distal axons or cells colabeled with subtype-specific markers relative to the total number of bipolar cell bodies in cross-sectional images from three or more retinas. Data were analyzed and presented as mean ± SEM using Prism software (GraphPad).

## Results

### Bipolar cell subtype-specific inducible Cre mouse lines

To generate inducible Cre knock-in mouse models at *Vsx1*, *Lhx3*, and *Lhx4* gene loci, a gene cassette containing adenovirus major late intron splicing acceptor sequence, P2A-CreER^T2^, and a rabbit β-globin polyadenylation signal (rBG polyA) was knocked in between exons 2 and 3 of *Vsx1* and *Lhx4*, and between exons 1 and 2 of *Lhx3*, for CreER^T2^ expression and rBG polyA transcription termination and gene knock-out (Fig. 1*C*,*D*). Whereas *Vsx1*CreER^T2^ homozygous knock-out mice survived postnatally, *Lhx3*CreER^T2^ and *Lhx4*CreER^T2^ homozygous knock-out mice were embryonic or perinatal lethal. Adult heterozygous mice from each Cre knock-in line were therefore used for subsequent experiments. These mice were crossed with a Cre-dependent channelrhodopsin-2 enhanced yellow fluorescent protein (ChR2EYFP) reporter mice to generate CreER^T2^;Ai32ChR2EYFP double-heterozygous mice for characterization of *Vsx1*, *Lhx3*, and *Lhx4* expression in retinal bipolar cell subtypes in developing and adult mouse retinas (Fig. 1*E*). ChR2 is a light-gated ion channel and integral membrane protein that mediates light-induced electrical activity, particularly in neurons (Fig. 1*F*). The expression of ChR2EYFP in bipolar cell subtypes optimizes visualization of cell morphology and allows manipulation of light-induced neuronal activity in bipolar cell subtypes (Madisen et al., 2012).

### Lineage tracing of *Vsx1*-, *Lhx3*-, and *Lhx4*-expressing bipolar cell subtypes in the adult mouse retina

Cell lineage tracing using inducible Cre models allows temporal control of genetic labeling in specific cells or tissues throughout development (Kretzschmar and Watt, 2012). Thus, to identify bipolar cell subtypes that express *Vsx1*, *Lhx3*, and *Lhx4* in the adult mouse retina, we induced Cre/loxP recombination in adult CreER^T2^;Ai32ChR2EYFP mice and performed immunolabeling on retinal tissues to assess ChR2EYFP reporter expression.

**Figure 2.**
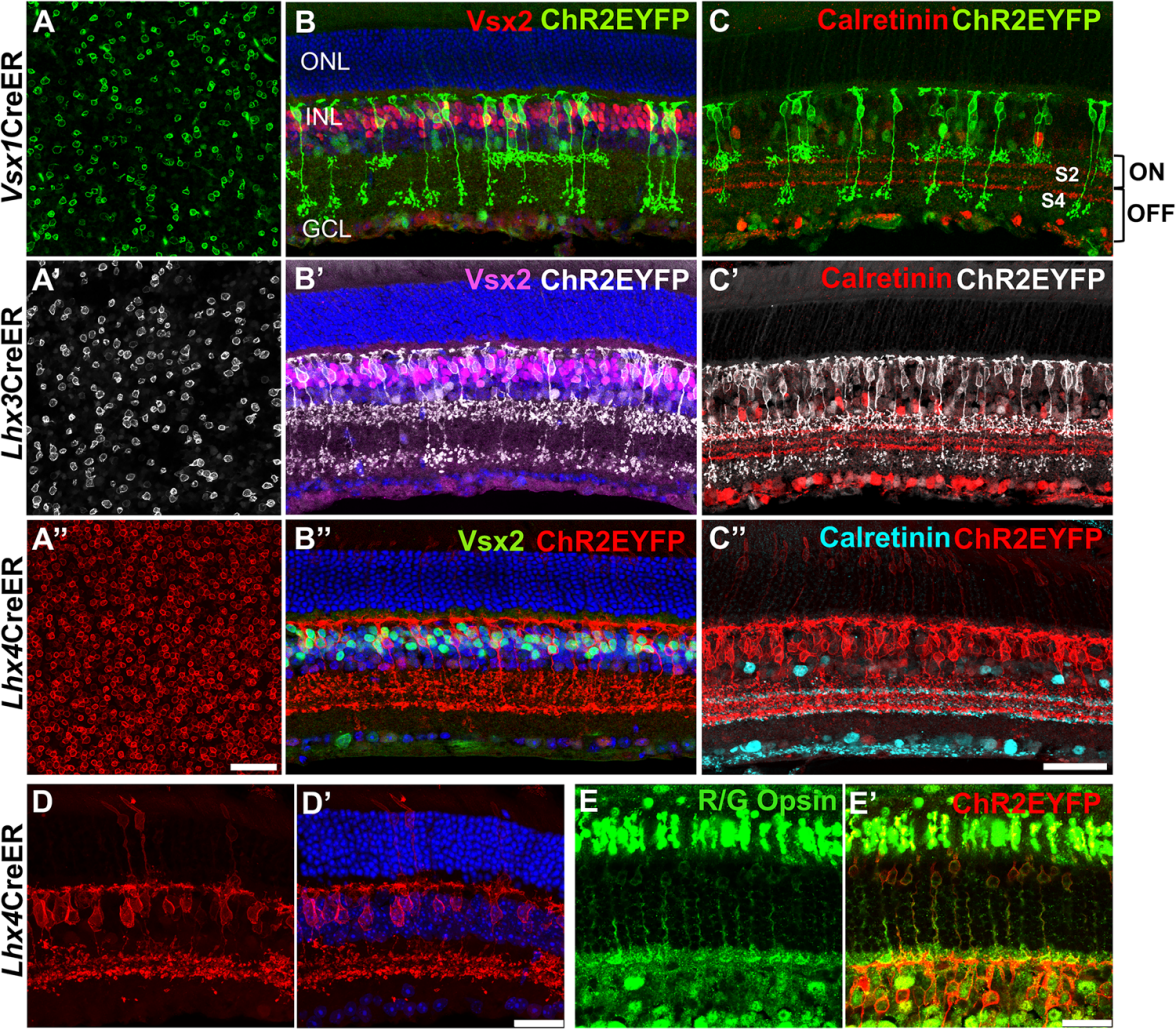
Lineage tracing of *Vsx1*, *Lhx3*, and *Lhx4* in ON and OFF bipolar cells and cone photoreceptor cells in the adult mouse retina. ***A***, Flat mount retinas showing ChR2EYFP expression in the cell membrane of *Vsx1*-, *Lhx3*-, and *Lhx4*-expressing retinal bipolar cell somas after 3× tamoxifen dosage. Scale bar in ***A″***, 50 µm. ***B***, Retinal cross sections showing colocalization of *Vsx1*-, *Lhx3*-, and *Lhx4*-expressing bipolar cells with pan bipolar cell marker Vsx2. ***C***, *Vsx1*-, *Lhx3*-, and *Lhx4*-expressing bipolar cell axons stratify in ON and OFF sublaminae layers in the IPL of the mouse retina. Scale bar (retina cross sections) in ***C″*** = 50 µm. ***D***, Maximum intensity projection retina cross-sectional images showing ChR2EYFP expression in *Lhx4*-expressing bipolar cells and photoreceptor cells. ***E***, Coimmunolabeling of *Lhx4*-expressing photoreceptor cells with red/green (R/G) opsin. Scale bar (***D***, ***E***), 25 µm.

Flat mount immunolabeling showed the expression of ChR2EYFP in the membranes of bipolar cells in adult *Vsx1*-, *Lhx3*-, and *Lhx4*CreER^T2^ retinas (Fig. 2*A*). *Lhx4*CreER^T2^ retinas showed higher density of ChR2EYFP-expressing bipolar cells (142,33 ± 445 cells/mm^2^) compared with *Lhx3*CreER^T2^ (2,754 ± 87 cells/mm^2^) and *Vsx1*CreER^T2^ (2,402 ± 158 cells/mm^2^) retinas given same dosage and concentration of tamoxifen (3×, 75 mg/kg). In retinal cross sections, cell bodies of CHR2EYFP-expressing bipolar cells colocalized with the pan bipolar cell marker CHX10/VSX2 (Burmeister et al., 1996) in the outer half of the inner nuclear layer (Fig. 2*B*). Coimmunolabeling of EYFP-expressing bipolar cells with calretinin also showed axon terminals of bipolar cell subtypes stratifying within ON and OFF sublamina layers in the inner plexiform layer (Ghosh et al., 2004), indicating *Vsx1*, *Lhx3*, and *Lhx4* expression in both ON and OFF cone bipolar cells (Fig. 2*C*). Notably, *Lhx4* expression was also detected in subsets of cone photoreceptor cells that coimmunolabeled with red/green opsin (Fig. 2*D*,*E*).

**Figure 3.**
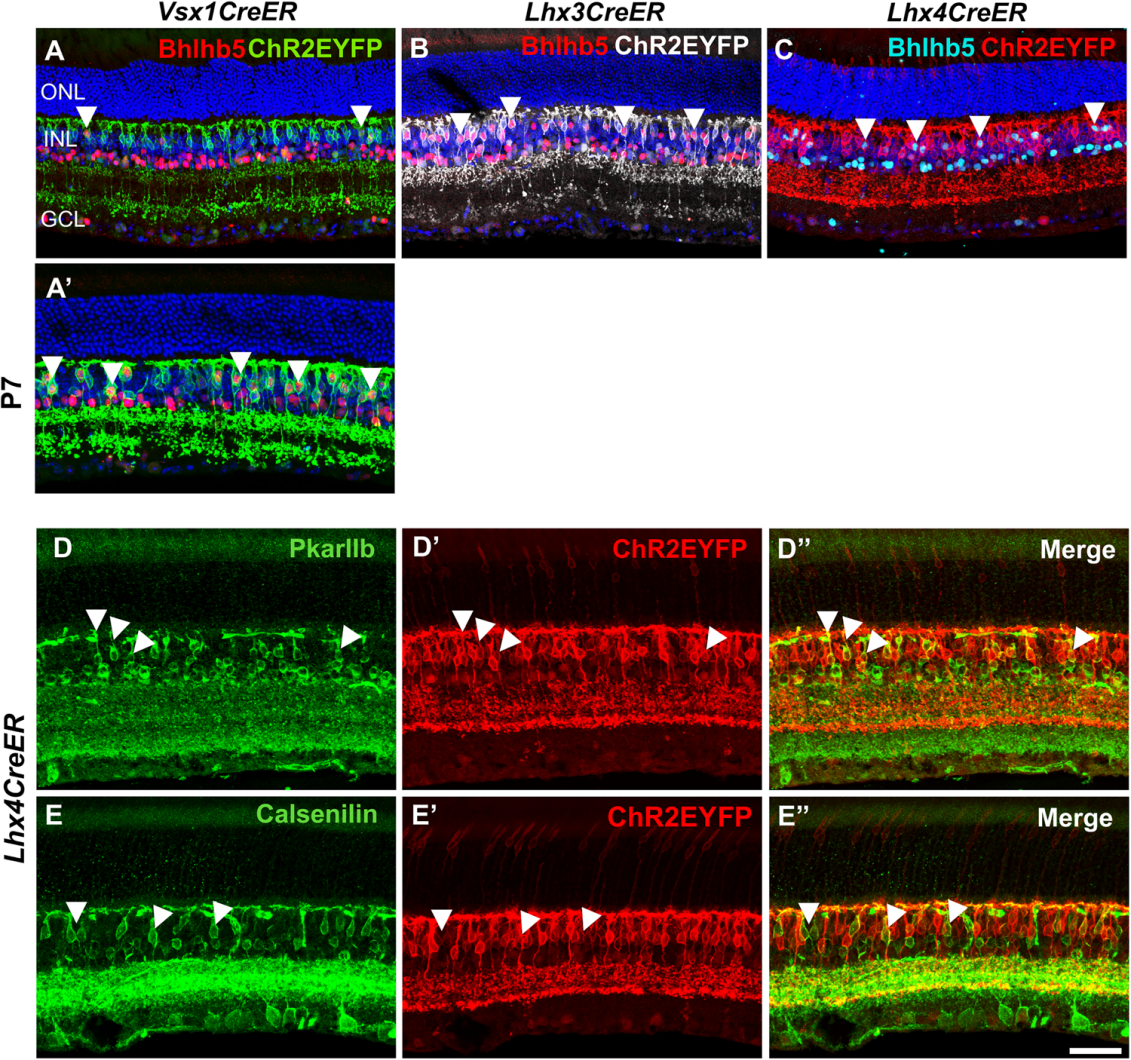
*Vsx1-*, *Lhx3*-, and *Lhx4*-expressing bipolar cells colocalize with OFF bipolar cell markers in the adult mouse retina. ***A–C***, Retina cross sections showing colocalization of type 2 bipolar cell marker Bhlhb5 in subsets of (***A***) *Vsx1*-, (***B***) *Lhx3*-, and (***C***) *Lhx4*-expressing bipolar cells. ***D***, ***E***, Coimmunolabeling of type 3 bipolar cell marker PkarIIb (***D***) and type 4 bipolar cell marker Calsenilin (***E***) with subsets of *Lhx4*-expressing bipolar cells. Scale bar, 50 µm.

The distinct stratification of ON and OFF bipolar cell axon terminals in *Vsx1*- and *Lhx3*-expressing bipolar cells enabled quantification of ON cone bipolar cells in *Vsx1*CreER^T2^ (59.9 ± 1.77%) and *Lhx3*CreER^T2^ (45.3 ± 4.51%) adult mouse retinas. Using the type 2 bipolar cell marker BHLHB5 (Feng et al., 2006), we also found that 12.7 ± 4% of EYFP-expressing bipolar cells in *Vsx1*CreER^T2^, 36.4 ± 3.48% of EYFP-expressing bipolar cells in *Lhx3*CreER^T2^, and 19.7 ± 1.36% of EYFP-expressing bipolar cells in *Lhx*4CreER^T2^ adult mouse retinas were type 2 bipolar cells (Fig. 3*A*–*C*). Given ∼40% *Vsx1*-expressing OFF bipolar cells with axon terminal stratification in S1, and ∼13% *Vsx1*-expressing type 2 OFF bipolar cells, there remains ∼27% nontype 2 OFF bipolar cells (type 1a) present in the *Vsx1*CreER^T2^ adult mouse retina. However, a substantially higher proportion of BHLHB5+ type 2 bipolar cells (40.3 ± 2.86%) were detected in *Vsx1*CreER^T2^ retinas when tamoxifen was administered at an earlier timepoint at P7 (Fig. 3*A*′), suggesting downregulation of *Vsx1* expression in type 2 bipolar cells in the adult mouse retina. Subsets of *Lhx4*-expressing bipolar cells also coimmunolabeled with the type 3b bipolar cell marker PRKAR2B (Mataruga et al., 2007; 23.1 ± 1.99%) and the type 4 bipolar cell marker calsenilin (Haverkamp et al., 2008; 22.9% ± 1.19%), indicating *Lhx4* expression in these subtypes (Fig. 3*D*,*E*). Notably, the observed bipolar cell densities in the adult CreER^T2^ mouse lines were less than those reported by Wässle et al. (2009). Since the detected density of EYFP-expressing bipolar cells depends on the level of Cre expression and tamoxifen dosage, the difference is likely due to lower tamoxifen dosage used in this study than in other studies (Madisen et al., 2012). Nevertheless, the high proportion of type 6 bipolar cells (45.3%) in *Lhx3*CreER^T2^ retinas and the relative distribution of type 2, 3b, and 4 bipolar cells (19.7, 23.1, and 22.9%, respectively) in *Lhx4*CreER^T2^ retinas were consistent with that reported in Wässle et al. (2009). Taken together, ChR2EYFP reporter expression shows that *Vsx1*-, *Lhx3*-, and *Lhx4*-expressing cells give rise to both ON and OFF cone bipolar cells and cone photoreceptor cells in the adult mouse retina.

### Sparse labeling and morphology of *Vsx1*-, *Lhx3*-, and *Lhx4*-expressing bipolar cell subtypes in the adult mouse retina

Sparse labeling is an essential method for visualizing and studying the morphology of individual cells. In an inducible Cre model, this method is achieved by varying and fine-tuning the concentration of the Cre-activating ligand. Thus, to sparse label and further characterize *Vsx1*-, *Lhx3*-, and *Lhx4*-expressing bipolar cell subtypes, tamoxifen concentration for Cre recombination in each Cre line was reduced and optimized to achieve reporter expression a few interspersed bipolar cell subtypes in the adult retina.

**Figure 4.**
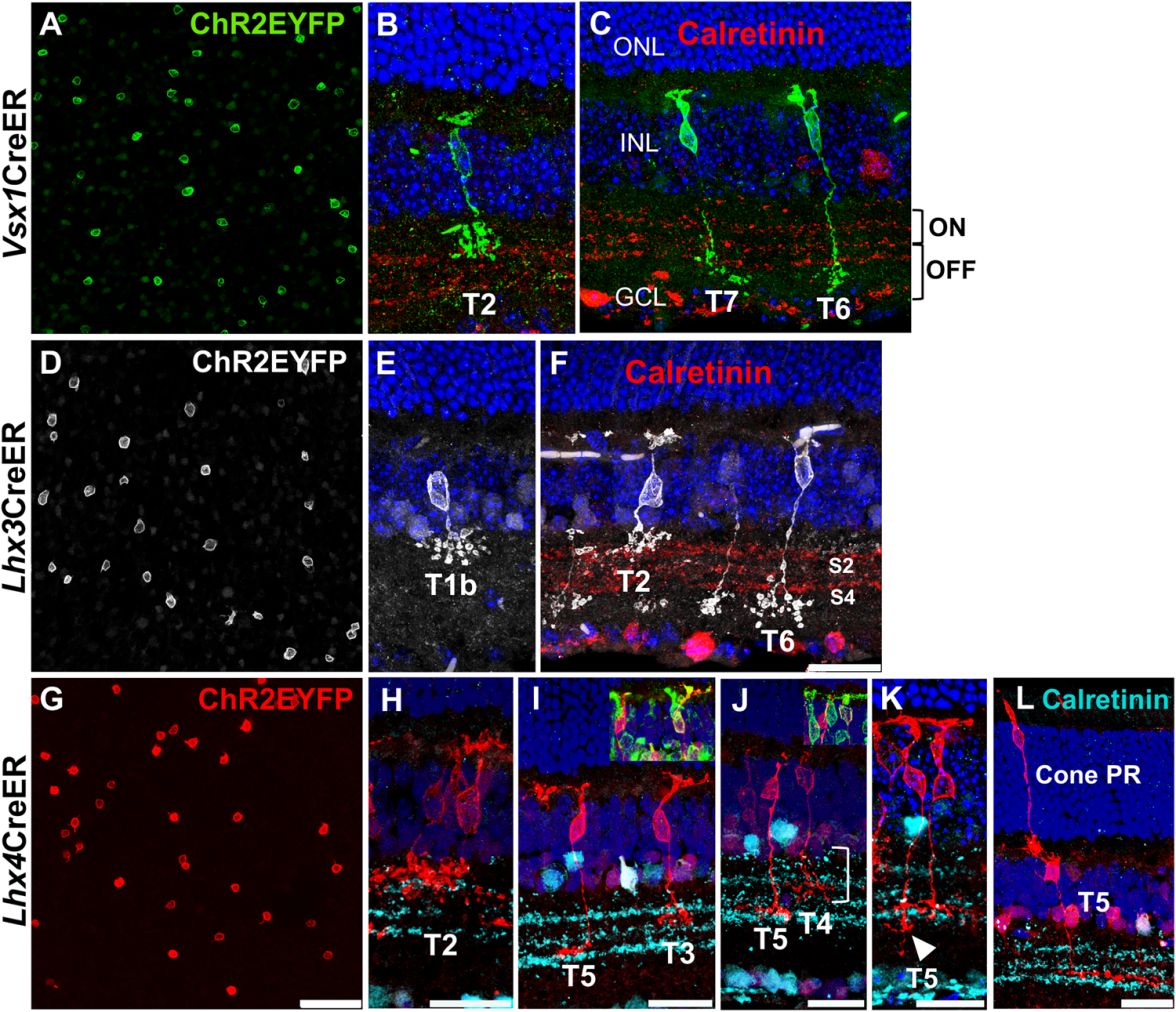
Sparse labeling of *Vsx1*-, *Lhx3*-, and *Lhx4*-expressing bipolar cells in the adult mouse retina. ***A–C***, Sparsely labeled *Vsx1*-expressing bipolar cells in a retinal flat mount and cross sections showing *Vsx1* expression in type 2 OFF and type 6 and 7 ON bipolar cells. ***D–F***, Sparsely labeled *Lhx3*-expressing bipolar cells in a retinal flat mount and cross sections showing *Lhx3* expression in type 1b and 2 OFF and type 6 ON bipolar cells. ***G–L***, Sparsely labeled *Lhx4*-expressing bipolar cells in a retinal flat mount and cross sections showing *Lhx4* expression in type 2, 3, and 4 OFF and type 5 ON bipolar cells and in a cone photoreceptor cell. Axon terminal stratification of some type 5 bipolar cells extends into sublaminae 4 (white arrowhead). Type 4 bipolar cells exhibit characteristic large axon arbor thickness (right bracket). Insets show colabeling of type 3 bipolar cell with PkarIIb and type 4 bipolar cell with calsenilin. Scale bar, 50 µm(flat mount)/25 µm (cross section).

Flat mount staining of sparsely labeled *Vsx1*-, *Lhx3*-, and *Lhx4*-expressing bipolar cell subtypes showed markedly reduced density of labeled bipolar cells (Fig. 4*A*,*D*,*G*). *Vsx1*-expressing bipolar cell subtypes was sparsely labeled with a single dose of 75 mg/kg tamoxifen. High magnification confocal imaging of sparsely labeled bipolar cells in retinal sections showed *Vsx1* expression in type 2, 6, and 7 bipolar cells (Fig. 4*B*,*C*). Axon terminals of type 2 bipolar cells stratified in S1 in the inner plexiform layer and were brush-like with dense varicosities (Ghosh et al., 2004). Both type 6 and type 7 *Vsx1*-expressing bipolar cells displayed axon terminals stratifying in S4; however, type 7 bipolar cells displayed more extensive terminal branching than type 6 bipolar cells. In addition, type 6 bipolar cells tended to have more elongated cell bodies compared with the relatively rounded cell bodies of type 7 bipolar cells.

*Lhx3*-expressing bipolar cell subtypes were sparsely labeled with a single dose of 40 mg/kg tamoxifen. High magnification confocal imaging of *Lhx3*-expressing bipolar cells in retinal sections showed *Lhx3* expression in type 2 and type 6 bipolar cells (Fig. 4*F*). *Lhx3* expression was also detected in type 1b bipolar cells (Fig. 4*E*), and ChR2EYFP labeling revealed their distinctive amacrine cell-like, unipolar cell body-axon morphology, as described by Shekhar et al. (2016) and Della Santina et al. (2016).

*Lhx4*CreERT^2^ mice required substantially lower tamoxifen concentrations—a single dose 15 mg/kg—to sparsely label *Lhx4*-expressing bipolar cell subtypes, highlighting the high density of *Lhx4*-expressing bipolar cells in the adult mouse retina. Further reduction of tamoxifen concentration (10 mg/kg) selectively labeled bipolar cell subtypes with axon terminals stratifying in S2 (type 3 and type 4). Morphology and axon terminal stratification of sparsely labeled *Lhx4*-expressing subtypes, however, showed *Lhx4* expression in type *2*, 3, 4, and 5 bipolar cells (Fig. 4*H*–*L*). Interestingly, type 2 bipolar cells were rarely detected in sparsely labeled *Lhx4*-expressing subtypes, suggesting relatively low *Lhx4* expression in type 2 bipolar cells in the adult retina. Type 3 and type 5 bipolar cells exhibited morphological variability; however, all variants had axon terminals stratifying in S2 and S3, respectively (Ghosh et al., 2004). Some type 5 bipolar cells had their axon terminals extending into S4, and axon terminals of some type 3 bipolar cells initiated branching in S1. Type 4 bipolar cells characteristically exhibited a larger arbor thickness, that is, a greater axon terminal branching depth extending across S1 and S2 (Ghosh et al., 2004; Tsukamoto and Omi, 2014). Since axon terminals of both type 3 and type 4 stratified in S2, a definitive identification relied on their coimmunolabeling with subtype-specific markers PkarIIb and Calsenilin (Mataruga et al., 2007; Haverkamp et al., 2008). Sparse labeling in *Lhx4*CreERT^2^ retinas also showed cone photoreceptors making synaptic contacts with cone bipolar cells in the outer plexiform layer (Fig. 4*L*).

### *Vsx1*, *Lhx3*, and *Lhx4* expression patterns during retinal bipolar cell genesis and differentiation

**Figure 5.**
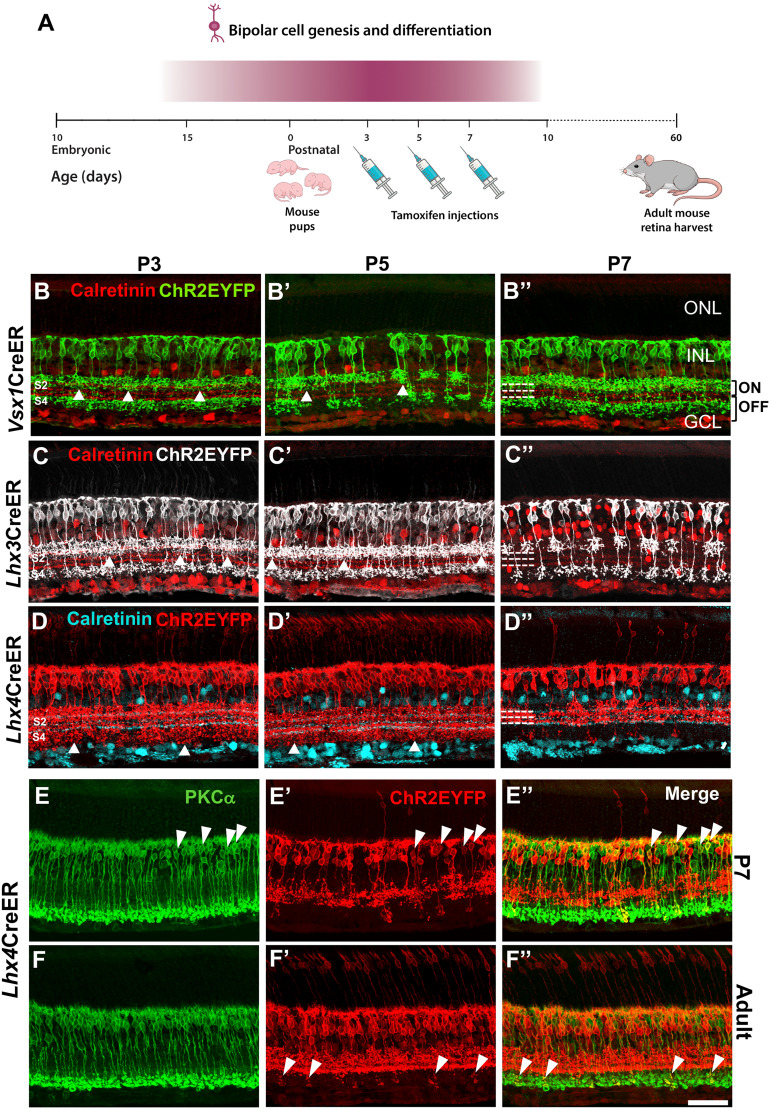
*Vsx1*, *Lhx3*, and *Lhx4* expression patterns during bipolar cell genesis and differentiation. ***A***, Schematic of experimental procedure involving tamoxifen injection in P3, P5, and P7 pups and retinal harvest for EYFP expression characterization at P60. ***B***, *Vsx1*-expressing bipolar cells in P3, P5, and P7 tamoxifen-injected mice showing axon terminal stratification in S1, S2, and S4 in the adult mouse retina. ***C***, *Lhx3*-expressing bipolar cells in P3, P5, and P7 tamoxifen-injected mice showing axon terminal stratification in S1, S2 (P3, P5), and S4 in the adult mouse retina. ***D***, *Lhx4*-expressing bipolar cells in P3, P5, and P7 tamoxifen-injected mice. *Lhx4* is expressed in bipolar cells with axon terminal stratification beyond S3 in P3 and P5 tamoxifen-injected mice, but mostly in bipolar cells with axon terminal stratification in S1, S2, and S3 in P7 tamoxifen-injected mice. ***E***, Coimmunolabeling of rod bipolar cell marker PKCα with a subset of *Lhx4*-expressing bipolar cells in the P7 tamoxifen-injected mouse retina. ***F***, Coimmunolabeling of rod bipolar cell marker PKCα with *Lhx4*-expressing axon terminals in the adult tamoxifen-injected mouse retina. Scale bar, 50 µm.

Peak genesis of bipolar cells in the mouse retina occurs within the first postnatal week alongside Müller glia and rod photoreceptors (Young, 1985). Expression of transcription factors in the retina during this period regulates the specification, differentiation, and subtype formation of bipolar cells (West and Cepko, 2022; Quainoo and Gan, 2025). However, transcription factors expression patterns in bipolar cell subtypes during this period of peak genesis remain unclear. To investigate this, we characterized *Vsx1*, *Lhx3*, and *Lhx4* expression in the retina during the first postnatal week. This was achieved through a single dose tamoxifen injection in *Vsx1*CreERT^2^, *Lhx3*CreERT^2^, and *Lhx4*CreERT^2^ mice on P3, P5, and P7, and characterization of EYFP expression in adult P60 retinas (Fig. 5*A*). The plasma half-life of tamoxifen is ∼12 h and complete degradation of tamoxifen occurs in ∼4–6 d after administration in mice (Valny et al., 2016). Thus, EYFP expression observed in P60 retinas reflects *Vsx1*, *Lhx3*, and *Lhx4* expression patterns in bipolar cell subtypes on the day of injection and 4–6 d postinjection.

Two possible outcomes were anticipated; that these transcription factors are expressed in specific bipolar cell subtypes at the onset of bipolar cell specification, as observed in the adult retina, or turned on and off in different bipolar cell subtypes at different developmental time points. In P3, P5, and P7 tamoxifen-injected mouse retinas, *Vsx1* expression was restricted to ON and OFF bipolar cells with axon terminal stratifications largely in S1 and S4 in the inner plexiform layer (Fig. 5*B*). However, *Vsx1* expression was also observed in bipolar cells with axon terminal stratifications in S2 in the P3, P5, and P7 tamoxifen-injected retinas, suggesting transient *Vsx1* expression in type 3 or type 4 bipolar cells during the first postnatal week. Similar patterns were observed in *Lhx3*CreER^T2^ retinas (Fig. 5*C*). Unlike in the adult retina, bipolar cells with axon terminal stratification in S2 were detected in P3- and P5-injected retinas. However, *Lhx3*-expressing bipolar cells in P7-injected retinas showed similar axon terminal stratifications as those observed in the adult mouse.

As in the adult retina, *Lhx4* was expressed in both photoreceptor cells and bipolar cells during the first postnatal week (Fig. 5*D*). However, in contrast to adults, *Lhx4* expression in P3- and P5-injected retinas was also detected in bipolar cells with axon terminals stratifying beyond S3. This indicates *Lhx4* expression in many ON cone bipolar cell subtypes and possibly rod bipolar cells during early postnatal development. In P7-injected retinas, *Lhx4* expression was largely restricted to bipolar cells with axon terminal stratification in S1, S2, and S3 as in adults. However, subsets of *Lhx4*-expressing bipolar cells in P7-intected retinas also coimmunolabeled with the rod bipolar cell marker PRKCA (Haverkamp and Wässle, 2000; Fig. 5*E*), indicating *Lhx4* expression in rod bipolar cells in early postnatal development. In the adult mouse retina, *Lhx4* expression was detected only in the axon terminals of a small number of rod bipolar cells, suggesting *Lhx4* downregulation or shutdown in rod bipolar cells in the course of development (Fig. 5*F*).

Taken together, *Vsx1*, *Lhx3*, and *Lhx4* show differential expression in bipolar cell subtypes at different time points during bipolar cell development. These findings suggest that temporally regulated transcription factor expression in bipolar cells plays a critical role in bipolar cell subtype differentiation and maintenance.

## Discussion

Here, we characterize the efficiency of inducible CreER^T2^ mouse models and trace the lineage of *Vsx1*-, *Lhx3*-, and *Lhx4*-expressing bipolar cell subtypes during their development in the postnatal and adult mouse retina. Our main findings show that *Vsx1*-expressing bipolar cells give rise to type 2 and 7, as well as type 6 bipolar cell subtypes. *Lhx3*-expressing bipolar cells give rise to type 1b, 2, and 6 bipolar cell subtypes, and *Lhx4*-expressing bipolar cells give rise to type 2, 3, 4, and 5 bipolar cell subtypes, as well as cone photoreceptor cells in the adult mouse retina. *Lhx4* is also shown to be widely expressed across multiple bipolar cell subtypes during bipolar cell specification and differentiation in the first postnatal week, with expression restricted to fewer subtypes in the adult retina. Similarly, *Vsx1* and *Lhx3* demonstrate slight variation in their expression pattern in bipolar cell subtypes during their development in the postnatal retina and in the adult mouse retina (Table 1).

**Table 1.** Summary of bipolar cell subtypes expressing CreER^T2^ in inducible Cre mouse lines

Inducible Cre mouse lines	Developmental stage	
	Adult P50	Postnatal P3–P7
*Vsx1*CreER^T2^	T1a? T2 T6 T7	T1a? T2 T3 T6 T7
*Lhx3*CreER^T2^	T1b T2 T6	T1b T2 T3 T6
*Lhx4*CreER^T2^	T2 T3 T4 T5, Cone PRs	T2 T3 T4 T5, RBCs, Cone PRs, ON Cone BCs terminating beyond S3

Tamoxifen injection in adult and postnatal inducible Cre mouse lines shows *Vsx1*CreER^T2^ expression in type 2, 6, and 7 bipolar cells and possibly in type 1a bipolar cells in the adult retina. *Lhx3*CreER^T2^ expression is also detected in type 1b, 2, and 6 bipolar cells in the adult retina. *Lhx4*CreER^T2^ expression is detected in type 2, 3, 4, and 5 bipolar cells and in cone photoreceptors in the adult retina. In the postnatal retina, *Vsx1*- and *Lhx3*- CreER^T2^ expression is additionally detected in type 3 bipolar cells, and *Lhx4*CreER^T2^ expression is detected in rod bipolar cells and ON cone bipolar cells terminating beyond sublaminae 3.

These inducible CreER^T2^ mouse models are essential tools for studying bipolar cell subtype development and function and augment the inadequate subtype-specific genetic tools available for the study of bipolar cells. Previous work has shown expression of *Vsx1*, *Lhx3*, and *Lhx4* in bipolar cell subtypes and supports the gene expression patterns observed in our study. *Vsx1* has been shown to be expressed in type 2, 6, and 7 bipolar cells, with transient or weak expression in type 1a and 3a bipolar cells (Shi et al., 2011; 2012; Shekhar et al., 2016). Knock-out of *Vsx1* results in incomplete terminal differentiation and impaired function of OFF cone bipolar cells and type 7 bipolar cells (Chow et al., 2004; Shi et al., 2011). However, the function of *Vsx1* in type 6 bipolar cells in the retina has not been well characterized. Our study shows *Vsx1* expression in OFF bipolar cells and in type 6 and 7 ON bipolar cells in the *Vsx1*CreER^T2^ mouse retina and thus provides a tool for structural and functional characterization of type 6 bipolar cells. The presence of BHLHB5 negative OFF bipolar cells in the *Vsx1*CreERT^2^ adult mouse retina in our study suggests *Vsx1* expression in type 1 bipolar cells. Axon stratification of subsets of bipolar cells in S2 in P3, P5, and P7 tamoxifen-injected *Vsx1*CreER^T2^ mice retinas also suggests transient expression of *Vsx1* in type 3 bipolar cells during the first postnatal week. This is consistent with previous studies and demonstrates *Vsx1* expression in noncanonical subtypes during bipolar cell development (Shi et al., 2012; Shekhar et al., 2016).

Balasubramanian et al. (2014) showed *Lhx3* expression in ON and OFF cone bipolar cells, and an scRNA-seq study by Shekhar et al. (2016) later showed this expression specifically in type 1b, 2, 3a, and 6 bipolar cells, as confirmed in *Lhx3*CreER^T2^ retinas. *Lhx3*CreER^T2^ adult mouse retinas showed the highest percent BHLHB5+ type 2 bipolar cells and type 6 bipolar cells, suggesting a role for *Lhx3* in the maintenance of these subtypes. *Lhx4* has also been reported to be expressed in several bipolar cell subtypes, specifically in types 2, 3, 4, and 5 bipolar cells, and transiently in rod bipolar cells (Dong et al., 2020). Shekhar et al. (2016) further showed weak *Lhx4* expression in type 1a, type 6, and rod bipolar cells in P17 mouse retinas. Similarly, *Lhx4* was expressed in bipolar cell subtypes that stratified beyond S3 following Cre induction in the postnatal *Lhx4*CreER^T2^ retina, in contrast to its expression in subtypes that stratified in S1, S2, and S3 following Cre induction in the adult retina. *Lhx4* expression is detected in 8 out of 15 identified bipolar cell subtypes in the adult retina and thus the high density of labeled bipolar cells in the adult *Lhx4*CreER^T2^ retina. Notably, *Lhx4* expression in the outer neuroblastic layer of the embryonic retina and inner nuclear layer of the postnatal retina (Blackshaw et al., 2004) suggests its role in differentiation of all, or a large subset of, bipolar cells in the developing retina. This is further supported by *Lhx4* expression in atypical ON bipolar cell subtypes during the first postnatal week and its later restriction to canonical type 2–5 bipolar cell subtypes in the adult mouse retina. *Lhx4* expression in rod bipolar cells during the first postnatal week also appears to decline as rod bipolar cells develop in the adult retina. These findings highlight the significance of temporally regulated transcription factor expression across bipolar cell subtypes during retinal development. It is plausible that several of these transcription factors are initially expressed in specified bipolar cells and selectively downregulated during subtype differentiation.

*Lhx4* has been shown to be expressed in nascent cone photoreceptor cells, and conditional knock-out of *Lhx4* results in defects in photoreceptor function (Buenaventura et al., 2019; Dong et al., 2020). Similarly, *Lhx4* expression was detected in photoreceptors in the first postnatal week and was maintained in a subset of cone photoreceptors in the adult retina. This expression pattern enables investigation of the structural and functional interactions between photoreceptors and bipolar cells using the *Lhx4*CreER^T2^ mouse model. Beyond the retina, *Vsx1* expression is detected in V2 interneuron precursors in the developing spinal cord (Francius et al., 2016), and *Lhx3* and *Lhx4* expression is also found in the developing pituitary gland, hindbrain, spinal cord, and lung (Li et al., 1994; Zhadanov et al., 1995; Weng et al., 2006). Thus, these inducible Cre models may also be used to assess and genetically manipulate mechanisms involved in the development of these tissues. Previous studies have demonstrated that diversity in visual information processing occurs in bipolar cells in a subtype-dependent manner (Ichinose et al., 2014; Hellmer et al., 2016). These Cre lines can therefore be used as tools to alter gene expression and subcellular functions in bipolar cell subtypes and provide further insight into their functional roles in visual information processing.

Studies show that ectopic expression of ChR in bipolar cells of 4–8-week-old mice induces no neurotoxicity (Ivanova and Pan, 2009; Doroudchi et al., 2011). Similarly, the expression of ChR2EYFP in bipolar cells of postnatal mice in our study did not cause any detectable structural changes in the bipolar cells when compared with bipolar cells expressing ChR2EYFP in the adult retina. This study characterizes the expression of *Vsx1*, *Lhx3*, and *Lhx4* in the adult mouse retina and compares with their expression in the postnatal retina during bipolar cell specification and subtype differentiation. Here, the use of ChR2EYFP as a reporter highlights bipolar cell subtype morphology and can be utilized to assess their electrophysiological activity. However, functional properties of these subtypes and their signaling interactions with photoreceptor cells remain to be tested in future studies. In summary, we demonstrate the efficiency of inducible Cre mouse lines that can be utilized for the study of bipolar cell subtypes and show the differential transcription factor expression patterns that occur and likely regulate bipolar cell subtype differentiation and maintenance.

## Synthesis

Reviewing Editor: Fabienne Poulain, University of South Carolina Decisions are customarily a result of the Reviewing Editor and the peer reviewers coming together and discussing their recommendations until a consensus is reached. When revisions are invited, a fact-based synthesis statement explaining their decision and outlining what is needed to prepare a revision will be listed below. The following reviewer(s) agreed to reveal their identity: NONE.

Bipolar cells in mouse retina have been the focus of many morphological, electrophysiological and molecular studies, leaidng to the consensus that different types of bipolar cells play distinct roles. However, the specific role of some bipolar types remains poorly understood. The present study uses inducible Cre lines under the promoter of Vsx1, Lhx3 and Lhx4 and crossing them with ChR2EYFP reporter mice to target bipolar cells in the developing and adult murine retina. The main finding is that these mouse lines show some subtype specific expression during development. The manuscript is accompanied by high quality Figures supporting the conclusions. However, the manuscript is tedious to read as it contains unnecessary repetitions, grammatical errors, unclarities and misleading wordings. The authors are encouraged to enlist the help of a neuroanatomist who is a native English speaker to assist with revisions because there are numerous errors in usage that need to be corrected. The following points need to be addressed before the manuscript can be considered for publication in eNeuro:

1. As the authors discuss, many of the results described in the present study are consistent with previous studies but it is not well explained how these new Cre lines will further our understanding of the functional role of bipolar cells.

2. The quantitative analysis is not explained. Please explain how the bipolar cell counts were made and how the bipolar proportions were calculated. How do these data compare to previous quantitative studies (e.g. Wässle et al., 2009, J. Neurosc.)?

3. The Introduction seems to be entirely about the mouse retina, though this is not stated. Bipolar cell diversity was recognized earlier in primates, and references to that work should be provided.

4. In the Methods section, it was unclear what was meant by [Author, University]. There is also a typo in the description of the section thickness.

5. There are numerous grammatical and wording errors. Below are a few examples (but there are many more):

> Except for the last 2 sentences, the entire first page needs to be rewritten.

> Page 4, last para: As intriguing as bipolar ... Please check this sentence

> Page 9: "In the outer segment of the inner nuclear layer" should be "in the outer [half]

> Page 10: "... made it possible to determine the percentage of ON [cone] bipolar ..." Assuming that rod bipolar cells are not included here?

> Page 11 second para, line 2 should read Figure 4

> Pages 12 and 13: "... we characterized Vsx1, Lhx3 and Lhx4 expression ... during the first postnatal week."; "...Lhx4 was expressed in ... bipolar cells during the first postnatal week." These statements are incorrect as the injections were done in the first postnatal week and the analysis was done in the adult retina.

> Page 14, last para, should read "... suggests Vsx1 expression in type 2 bipolar cells"

> Please use standard terms to describe axon stratification. On p 10 and 11 in the Results section, the word "laminate" is used incorrectly twice. "laminating" on p 10 seems to mean "terminating". It is unclear what "more laminated" means on p 11. A summary of the results in a table would be helpful.

> In the Discussion, the authors use "temporal" to describe the light responses, but I think they mean "transient". The word "together" is used incorrectly at the end.

6. References:

> The authors state that all 15 types of bipolar cells make distinct types of connections but do not provide a reference.

> Quainoo and Gan 2025: This reference is incomplete

7. The clarity of the manuscript would be greatly improved, if all panels had labels, like those in Figure 2. A summary diagram with a timeline would also be very useful.

8. Details about ethical approval for animal use were not provided.
